# Empyema of the Antrum

**Published:** 1888-07

**Authors:** G. W. Watson


ARTICLE IV.

### EMPYEMA OF THE ANTRUM.

BY G. W. WATSON.

CASE I.—Some 18 months ago a gentleman called on me complaining of toothache. The tooth referred to had quite a superficial cavity in it, which I filled; but on examining his mouth, I found that he had several abscessed teeth in the upper jaw, and noticed a peculiar odour, which

I knew to associate with antral or nasal trouble. I asked him if there was anything wrong with him. He acknowledged that he had been suffering from a discharge of pus from the right nostril, accompanied by severe frontal headache for 18 months, and had been under treatment by an expert, who had, however, done no good. He had also consulted two experts in London, one of whom diagnosed the case to be one of ozæna; the other said there was a tumour in the frontal sinus, which was the origin of the trouble, and would require to be removed. After a careful examination, I came to the conclusion that it was a case of antral empyema, and told him that I thought he could be put all right if he allowed me to remove all the diseased teeth in the upper jaw and make an opening into the antrum. After having consulted his friends and medical attendant, he agreed to be operated on. The condition of patient previous to operation was this :—

Great depression of spirits, severe frontal headache, which was almost constant, the parts over the frontal sinus and superciliary ridges were painful to the touch, and there was a copious discharge of a muco purulent fluid from right nostril, so much so that if he stooped a drop would fall from his nose; while the odour coming from him was perfectly overpowering, and the sense of smell had almost entirely gone. The patient attributed his condition to a severe chill caught while travelling to London. In presence of his medical attendant, I gave the patient a full dose of nitrous oxide, and removed the first upper bicuspid and molar on the right side, and two teeth on other side also, and made a free opening into the antrum through the socket of the first pre-molar, which I found communicated with the antrum. As a confirmation of my diagnosis, a flow of pus was obtained from the antrum on opening into it, and on syringing the fluid passed quite freely through the middle meatus into the nose. I moulded a portion of gutta-percha round a piece of iron binding wire, and used this as a temporary plug for the opening into antrum, till a proper silver drainage tube could be made. The patient was instructed to wash out the antrum thrice daily with a hot saturated solution of boracic acid. In the course of a fortnight the frontal headache had almost gone, and patient felt very much better in every respect. The patient was told that in all probability it would be a year before he was all right again, and such was the case. In two or three months the pain in region of frontal sinus had entirely disappeared, recurring again slightly later on as a result of nasal catarrh. The syringing of antrum daily, either with solution of zinc chloride or boracic acid, gradually brought the mucus membrane into a healthy condition and a subsidence of the discharge, and about the end of the year of treatment I removed the drainage tube, and the opening into the antrum closed up in the course of two or three days. At present time, six months since the completion of case, the patient is in perfect health. I have no doubt that the abscessed teeth were the origin of all those symptoms, causing, in the first place, irritation, then inflammation and alteration of secretion in mucus membrane of antrum, and the extension of this inflammatory condition to the nasal membrane, both from continuity of surface and the passage over it of the irritating purulent discharge from the antrum. The pain experienced in the region of frontal sinus, and which no doubt misled the London expert in his diagnosis, was, I have no doubt, purely reflex. The most instructive part of the case is, that none of the medical gentlemen ever suspected or even looked at the teeth to see what condition they were in.

CASE 2. History.—The patient was a clerk, æt. 42, of rather a delicate appearance. At beginning of last July, he was suffering very much from toothache in the upper jaw, and a feeling as if he had caught cold. He went one evening to a flower show, returning home on the top of a tramcar. Next day he had a severe rigour, was very sick, and vomited frequently, the illness culminating in nasal and gastric catarrh. This illness lasted for about a mouth, during which he was attended by his doctor. The right eye became swollen, and was useless for three or four weeks, and the right nostril was blocked by secretion, and continued so till he came into my hands some months later. His doctor took him to an expert in August, as no good had been obtained from douching the nasal cavity. He diagnosed the case to be one of ozæna, and said there was also diseased bones in right nasal cavity, and recommended patient to continue syringing the nose with boric solution. He was sent to the country for change of air, and remained away about a month. On his return it was found that matters were still in the same condition, the right nostril quite closed, and a constant discharge of a muco-purulent fluid, accompanied by the usual powerful odour. A week or two after this, the expert noticed a slight bulge of the cheek on right side, and thinking the antrum might have something to do with the condition of matters, told him to call on me. This was on October 22nd. In passing, I may say that the patient himself suggested to the doctor that the teeth might have something to do with it, as they had been so troublesome; but he always pooh-poohed the idea. On examining the patient, I saw it was a case of antral empyema, consequent on the presence in the jaw of abscessed teeth. Next day, in presence of his doctor, I gave patient a deep dose of nitrous oxide, and removed the second upper bicuspid and first and second molars on the right side, and on a subsequent occasion two teeth on left side, and made a free opening into the antrum through the socket of the second premolar. On attempting to syringe out the antrum, I found that for a considerable time nothing would come; but eventually, after using very great force with the ball syringe, a tablespoonful of caseous-looking purulent secretion came away from nostril, some of it of a blackish colour from pigmentation. In spite of this only a small stream of the injected fluid could be forced through the antrum and nose, and he could not breathe through the nostril. However, at eight o'clock the same evening, he managed to do so for the first time for months. On passing a probe into the antrum, I could detect bare bone on its nasal wall. A silver drainage tube was put through, the opening into antrum being kept in position by a piece of thin gold wire round the first pre-molar, and patient was instructed to syringe through the tube with saturated solutioo of boric acid thrice daily, keeping the tube closed with a plug of cotton wool in the intervals. Next day a piece of dead bone about the size of a small finger nail came away from the nostril while syringing, and since then several small fragments have been removed by the same means.

After using boric solution for three or four weeks with great advantage, I told him to use zinc chloride, two grains to the ounce to begin with twice daily, and the boric solution once; under which treatment and the use of tonics he has rapidly improved, and at the present time (March) the secretion has become very much less, the patient being able to do with one handkerchief per day, when he used to require a dozen. The nostril is quite free and there is no indication of diseased bone, the odour also being very much less. I took a photo-micrograph of a small portion of the caseous pus that came away after opening into and syringing the antrum. This I now hand round. It swarms with micrococci and bacilli. As the case went on, I made periodical microscopical examination of the secretion, antrum and nose, and found that there were fewer of these organisms each time I examined, and also less of the pus cell, and more of the normal mucous elements present. This patient was shown to the members.

I may say that I have had other two cases during the year—one where the disease had been going on for five years, the true origin of which, however, had been overlooked by several medical practitioners whom he had consulted. He was put all right, after being treated for about three months. The other case was the work of an unqualified practitioner, filling a canine tooth over an exposed pulp.

In conclusion, I would urge upon all who come across such cases the thorough examination of the mouth, the removal of all abscessed teeth, and the filling of all those that require it.—*The Dental Record.*

